# Supplementary material for: Genomic malaria surveillance of antenatal care users detects reduced transmission following elimination interventions in Mozambique
Source: Nat Commun. 2024 Mar 16;15:2402. doi: 10.1038/s41467-024-46535-x (PMC10944499; doi:10.1038/s41467-024-46535-x)
Supplement: Supplementary file 1 — Supplementary Information [file 41467_2024_46535_MOESM1_ESM.pdf]

Supplementary material to: Brokhattingen, N., Matambisso, G., da Silva, C., *et al.* Genomic malaria surveillance of antenatal care users detects reduced transmission following elimination interventions in Mozambique.

Table of contents

Supplementary methods 3

Study area 3

DNA extraction 3

Library preparation 4

Supplementary Results 5

Supplementary Figure 1. MOIRE estimates of *P. falciparum* multiplicity of infection compared to naive estimates 5

Supplementary Figure 2. *P. falciparum* parasite densities in first antenatal care users across the study period by area 6

Supplementary Figure 3. Effective multiplicity of infection in first antenatal care users by area and year 7

Supplementary Figure 4. Temporal trends in multiplicity of infection estimated naively and without adjusting for intra-host relatedness among first antenatal care users and children 8

Supplementary Figure 5. Comparison between *P. falciparum* genetic metrics and parasite rates in first antenatal care users 9

Supplementary Figure 6. Comparison between *P. falciparum* genetic metrics and parasite rates in children in Magude 10

Supplementary Figure 7. Genetic relatedness (pairwise identity by descent) within and between areas for infections in first antenatal care users and children 11

Supplementary Table 1. Factors associated with *Plasmodium falciparum* multiplicity of infection estimated naively and without adjusting for intra-host relatedness among first antenatal care users and children 12

Supplementary Table 2. Effect of gravidity on intra-host genetic diversity in first antenatal care users by area 13

Supplementary Table 3. Temporal trends in intra-host genetic diversity first antenatal care users in each area 14

Supplementary Table 4. Differences in temporal trends in Magude genetic intra-host diversity between first antenatal care users and children 15

Supplementary table 5. Correlation between trends in genetic intra-host diversity and *P. falciparum* parasite rates in first antenatal care users by area 16

Supplementary table 6. Correlation between trends in genetic intra-host diversity and *P. falciparum* parasite rates in children in Magude 17

Supplementary Table 7. Expected heterozygosity in first antenatal care users by area and years 18

Supplementary Table 8. Expected heterozygosity in first antenatal care users and children 19

Supplementary Table 9. Pairwise relatedness (identity by descent) within and between areas for first antenatal care users 20

Supplementary Table 10. Pairwise relatedness (identity by descent) between infections in pregnant women attending antenatal care and children overlapping in time 21

Supplementary methods

Study area

In Manhiça District, transmission is generally low, with rapid diagnostic test (RDT) *P. falciparum* parasite rates in children declining from 5% to 0% (to 0·3% by quantitative polymerase chain reaction [qPCR]) during the study period.[1] Ilha Josina, located 45 km northeast of Manhiça village, is a river island with historically high transmission. During the study, RDT *P.falciparum* parasite rates in children in Ilha Josina declined from 31% to 22%.[1] Magude district, located west of Manhiça district, was subject to a package of interventions in 2015-2018 including mass drug administration with dihydroartemisinin- piperaquine and IRS, resulting in a 85% reduction of in all-age *P.falciparum* parasite rates by RDT.[2] During this study, RDT *Pf* parasite rates in children declined from 3% to 1% in the area.[1]

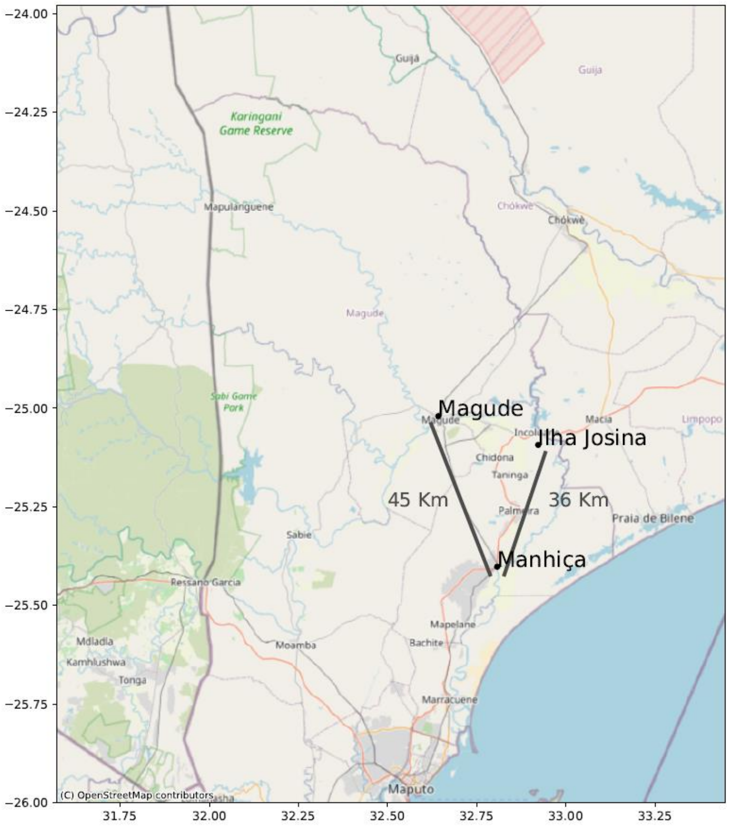

Map of study sites in southern Mozambique. Distances between Manhiça and Magude and Ilha Josina indicated. Map produced using OpenStreetMap data, available under the Open Database License.

DNA extraction

6 mm (~20 µl blood) discs were cut from each DBS into 96-well deep well plates with a manual puncher.1 mL Phosphate Buffered Saline (PBS, Sigma-Aldrich®) -Tween (0·05% Tween-20) was added per well, and the plate was incubated at 4°C overnight. The next day, liquid was aspirated from wells, followed by addition of 1 mL PBS and incubation at 4°C for a minimum of 30 min. After incubation, the liquid was aspirated, 150 µl 10% Chelex-100 (Bio-Rad®) was added per well, and the plate was placed in a water bath at 95°C for 10 minutes. Finally, extracted DNA was centrifuged at 20,913 x g for 5 min, and the supernatant was transferred to Micronics (Micronics®) tubes and stored at 4°C.
A standard curve was made from a dilution series of known parasite concentrations, from which the concentration in the samples could be extrapolated. A negative control with no parasite DNA was included in all reactions.

Library preparation

Targeted genomic regions of interest in the extracted parasite DNA were amplified in a multiplex PCR reaction (6 µl genomic DNA in a 10 µl reaction) and Illumina-specific adaptors were added. Two different protocols were used, depending on parasitemia. For samples with ≥100 parasite/µl, 10 min initial denaturation at 95°C was followed by 15 cycles of 15 seconds denaturation at 98°C with 3°C/s ramping, and 5 min annealing at 60°C with 2°C/s ramping. For samples with <100 parasite/µl, 10 min initial denaturation at 95°C was followed by 20 cycles of 15 seconds denaturation at 98°C with 3°C/s ramping, and 5 min annealing at 60°C with 2°C/s ramping. Subsequently, 2 µl STOP buffer and 10 µl 1XTE buffer was added to the multiplex PCR products. Next, bead-based purification was performed to remove primer dimers by adding SPRI (Solid Phase Reversible Immobilization) beads in a 1:1.3 sample:beads volume ratio, followed by two washes with 70% freshly prepared ethanol, and resuspension in 10 µl 1XTE buffer. A 10-min digestion reaction at 37°C further removed small DNA fragments from the samples, followed by another 1.3X bead-based purification. Then a secondary indexing PCR reaction was used to add sample-specific barcode sequences at both ends of the purified amplicons. 10 min initial denaturation at 95°C, was followed by 15 cycles of 15 seconds denaturation at 98°C with 3°C/s ramping, and 1 min and 15 seconds annealing at 60°C with 2°C/s ramping. The quality of the amplified DNA was checked on a Bioanalzyer capillary electrophoresis system (Agilent technologies, Santa Clara, CA, USA) before pooling the library. A final 1:1 sample:SPRI beads volume ratio purification was performed, before the library was run on a 2.5% agarose gel in TBE buffer with 1X SYBRsafe at 140V for 1 hour. The 400 bp band was excised, DNA extracted using Monarch® Genomic DNA Purification kit (New England Biolabs, Ipswich, MA, USA), and the quality of the library was checked on the Bioanalyzer. Mock DBS samples with known *Pf* concentrations were used as positive controls, while H_2O was used as negative control, and were included in all plates. All multiplex PCR, digestion, and indexing PCR reagents were from Paragon Genomics (Paragon Genomics Inc, CA, USA). The purified library pool was sequenced with 150 bp paired-end clusters on an Illumina NextSeq 550 instrument (Illumina, San Diego, CA, USA).

Supplementary Results

Supplementary Figure 1. MOIRE estimates of *P. falciparum* multiplicity of infection compared to naive estimates

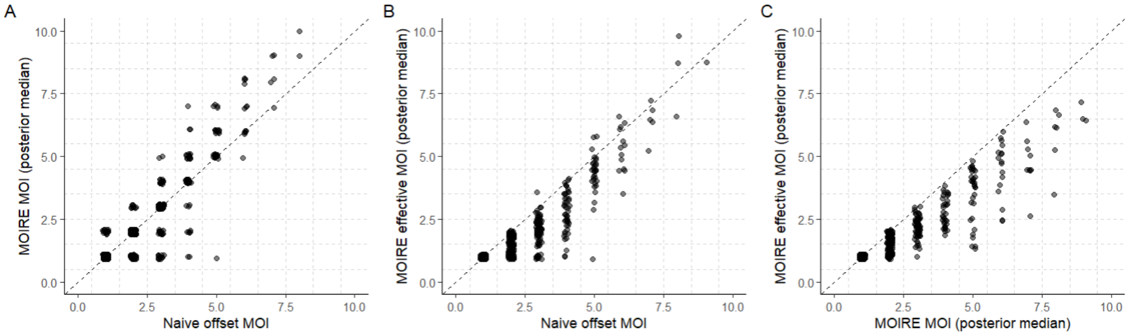

Estimates of multiplicity of infection (MOI) per sample (N= 382) is compared between **A.** naive offset MOI, i.e., the second highest number of distinct alleles observed in one locus, and MOI estimated using MOIRE (R package), **B.** naive offset MOI, i.e., the second highest number of distinct alleles observed in one locus, and effective MOI (eMOI), i.e., adjusted for intra-host relatedness between parasite strains, also estimated with MOIRE, and **C.** MOI and eMOI, both computed with MOIRE.

Supplementary Figure 2. ***P. falciparum*** **parasite densities in first antenatal care users across the study period by area**

No significant trends in parasite densities of infections are observed over time in each area (p=0.70) in a linear regression in pregnant women attending their first antenatal care visit. n=120 in Magude, n=64 in Manhiça, n=105 in Ilha Josina.

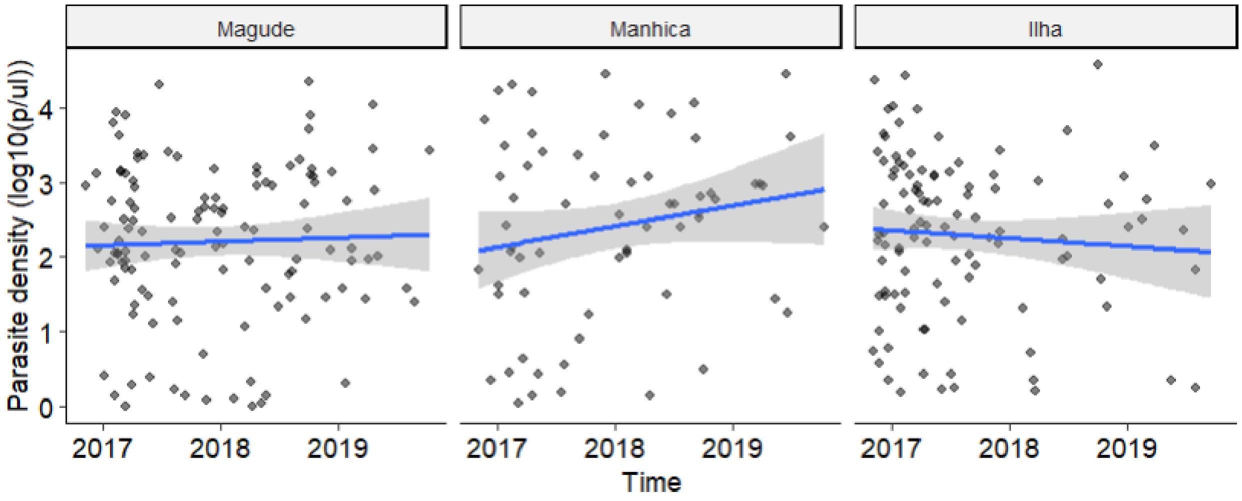

Supplementary Figure 3. Effective multiplicity of infection in first antenatal care users by area and year

Distribution of samples with effective multiplicity of infection (eMOI) by area and year. eMOI, estimated with *MOIRE* (R package[3]), can be interpreted as the multiplicity of infection adjusted for intra-host diversity between different parasite strains. n=120 in Magude, n=64 in Manhiça, n=105 in Ilha Josina.

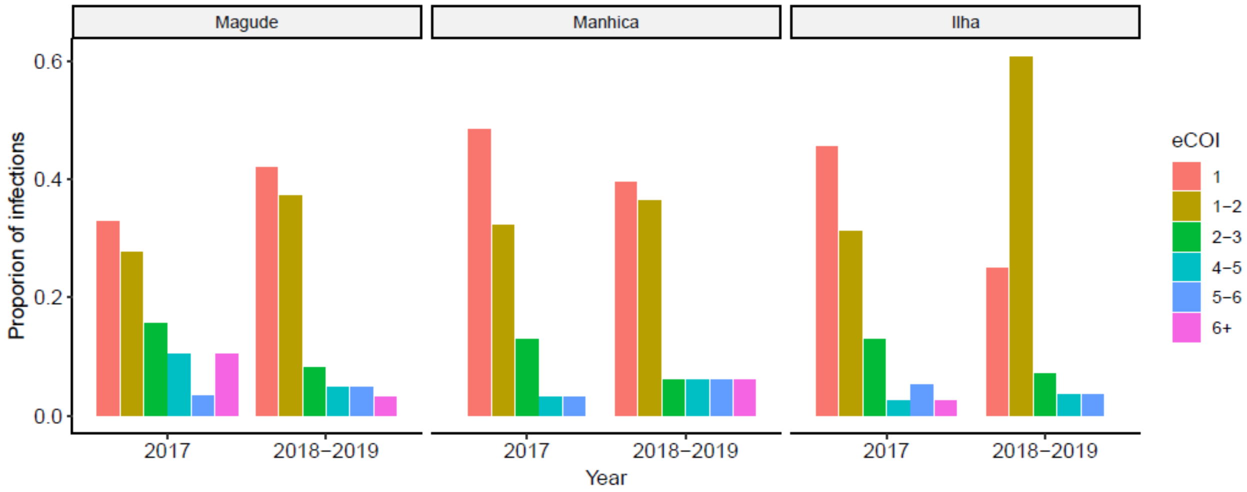

Supplementary Figure 4. Temporal trends in multiplicity of infection estimated naively and without adjusting for intra-host relatedness among first antenatal care users and children

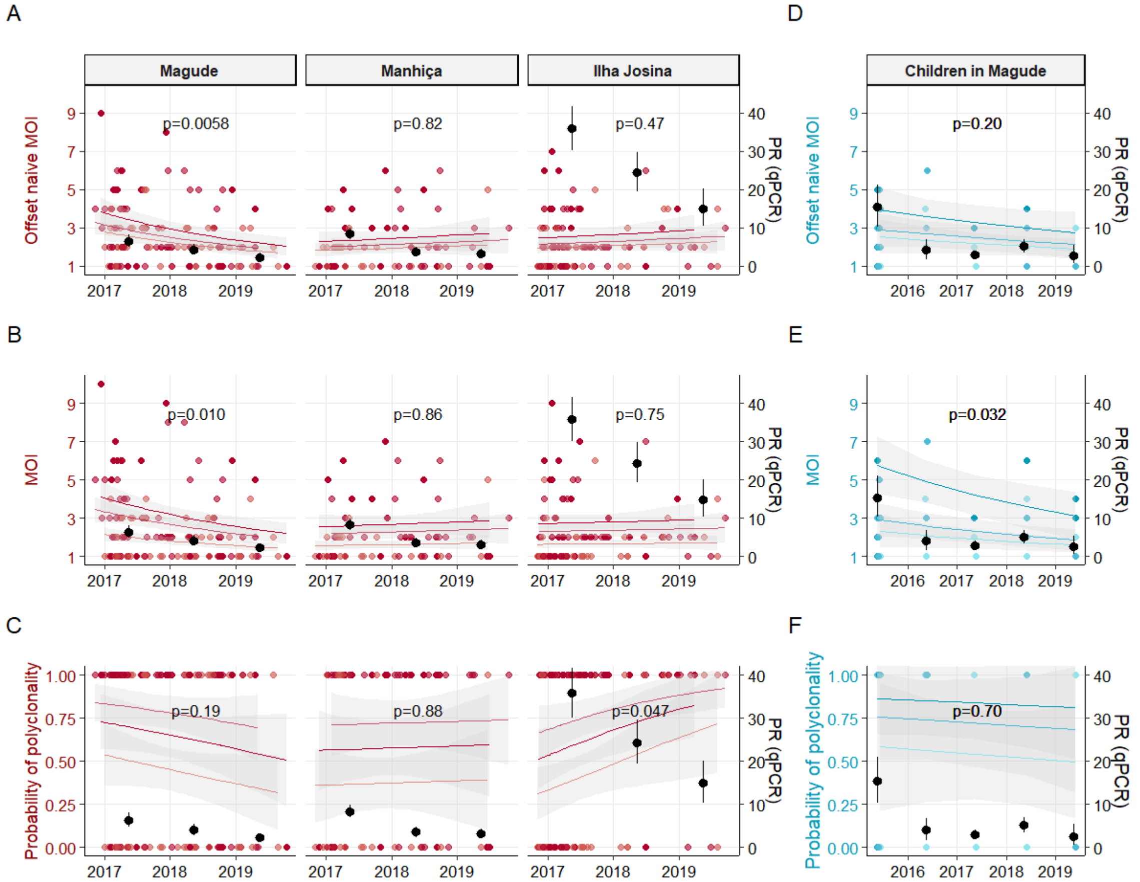

Intra-host genetic diversity over time in pregnant women attending their first antenatal care (ANC) visit (in red) by area (N=120 in Magude, n=64 in Manhiça, n=105 in Ilha Josina), and children aged 2-10 years old from the community (blue, n=47). The shade of red/blue indicates parasite density, with darker shade reflecting higher densities. Black dots represent *Plasmodium falciparum* (*Pf*) parasite rates (PR) by qPCR in the same population with 95% confidence intervals (CI) bars. **A.** Naive offset multiplicity of infection (MOI, defined as the second highest number of distinct alleles observed within one sample) in pregnant women attending their first ANC visit by area. P-values for temporal trend of naive offset MOI in Poisson regression adjusted for parasitemia (*Pf* parasites/µl) with 95% confidence interval (CI) bands. **B.** MOI estimated using MOIRE (R package) in Poisson regression adjusted for parasitemia with 95% CI bands. **C.** Monoclonal and polyclonal (MOI>1) infections in pregnant women at ANC by area in a logistic regression adjusted for parasite density with 95% CI bands. **D-F.** naive offset MOI, MOIRE-estimated MOI, and polyclonality in children aged 2-10 years from Magude, estimated with Poisson and logistic regressions similar to **A-C**. P-values in all graphs are for the temporal trend of the given metric in the regression (F-test). Adjusted for multiple testing using the Benjamin-Hochberg method, a p-value of less than 0.0062 indicates statistical significance.

Supplementary Figure 5. Comparison between *P. falciparum* genetic metrics and parasite rates in first antenatal care users

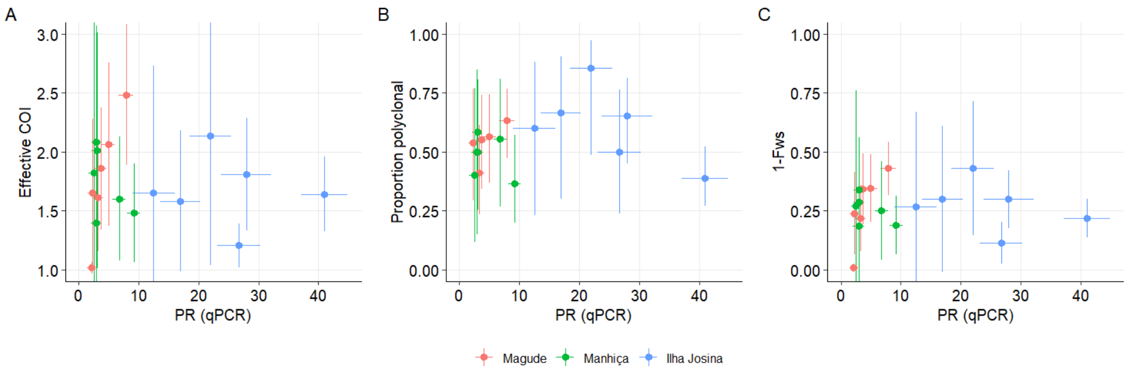

P. falciparum parasite rates (PR) by quantitative polymerase chain reaction (qPCR) is compared with **A.** effective multiplicity of infection (eMOI), **B.** proportion of infections that are polyclonal, and **C.** 1-Fws in pregnant women at their first antenatal care visit. Each dot represents estimates for one year with 95% confidence intervals. N=120 in Magude, n=64 in Manhiça, n=105 in Ilha Josina.

Supplementary Figure 6. Comparison between *P. falciparum* genetic metrics and parasite rates in children in Magude

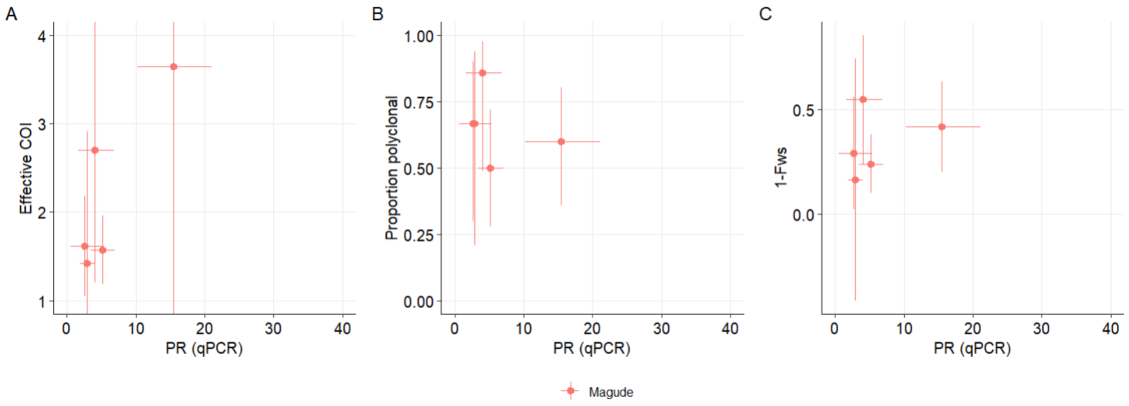

P. falciparum parasite rates (PR) by quantitative polymerase chain reaction (qPCR) in children aged 2-10 years sampled in household surveys are compared with **A.** effective multiplicity of infection (eMOI), **B.** proportion of infections that are polyclonal, and **C.** 1-Fws in pregnant women at their first antenatal care visit. Each dot represents estimates for one year with 95% confidence intervals. N=47.

Supplementary Figure 7. Genetic relatedness (pairwise identity by descent) within and between areas for infections in first antenatal care users and children

A. Mean identity by descent (IBD) among infections sampled from pregnant women at first antenatal care (ANC) visit (n=289, n=83,521 comparisons) within and between areas. IBD is defined as the proportion of the genome shared between infections because of recent shared ancestry, was estimated with *Dcifer* (R package)[4]. It uses naive MOI and population allele frequencies to account for the fact that alleles present in both infections may match by chance. Multiallelic data from unphased polyclonal infections are included. Overall mean IBD between ANC infection pairs was 0.026 (96% CI: 0.022;0.033). Mean pairwise IBD within Magude was significantly higher than what would be expected to be observed at random (p=0.002) **B.** Pairwise IBD between 39 samples from pregnant women at ANC and 31 samples from children in the community that overlap temporally (15 April-30 June, 2017-2019, a total of 4,900 comparisons). Each square represents a sample-sample comparison.

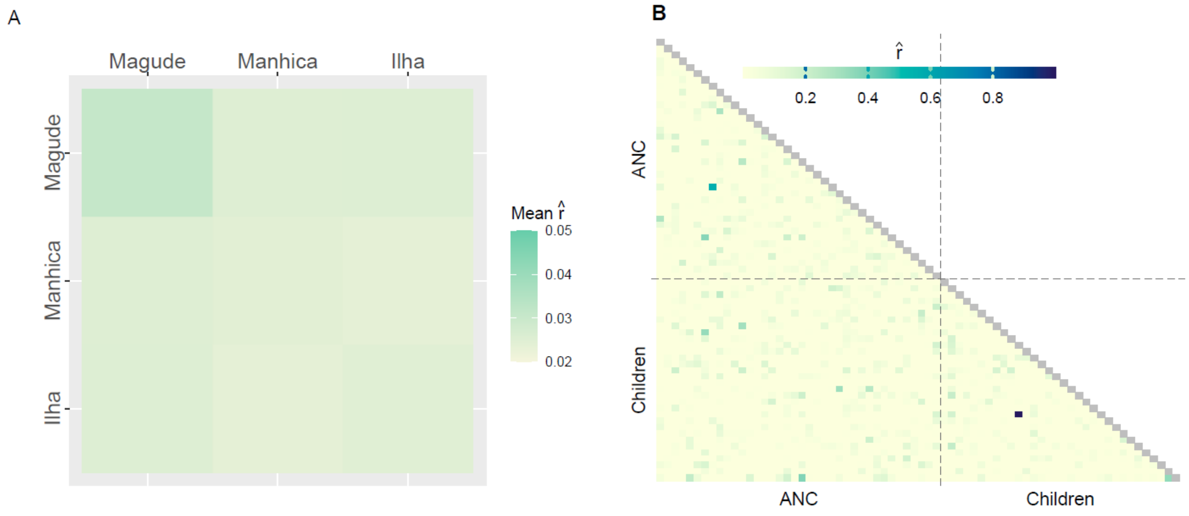

Supplementary Table 1. Factors associated with *Plasmodium falciparum* multiplicity of infection estimated naively and without adjusting for intra-host relatedness among first antenatal care users and children

First ANC users	Naive offset MOI (95% CI)	p	p*	MOI (95% CI)	p	p*	% polyclonal (95% CI)	p	p*
**All** (n=289)	2.37 (2.20;2.55)			2.37 (2.19;2.55)			60.2 (54.5;65.7)		
**Year**		0.46	0.16		0.48	0.35		0.54	0.17
2017 (n=166)	2.43 (2.20;2.67)			2.42 (2.19;2.67)			58.4 (50.5;65.9)		
2018-2019 (n=123)	2.29 (1.78;2.94)			2.29 (1.78;2.94)			62.6 (53.4;71.0)		
Change per year	-0.048 (-0.15;0.048)	0.33	0.039	-0.057 (-0.15;0.039)	0.25	0.077	NA	NA	0.11
**Area**		0.13	0.053		0.20	0.13		0.71	0.21
Magude (n=120)	2.58 (2.31;2.88)			2.56 (2.28;2.86)			62.5 (53.2;71.0)		
Manhiça (n=64)	2.16 (1.57;2.93)			2.20 (1.61;2.99)			56.2 (43.3;68.4)		
Ilha Josina (n=105)	2.26 (1.70;2.98)			2.25 (1.69;2.97)			60.0 (50.0;69.3)		
**Parasite density**		0.041	0.039		**2.2e-07**	**2.6e-07**		**1.0e-04**	**3.2e-06**
<100 p/µL (n=103)	2.11 (1.84;2.49)			1.74 (1.50;2.01)			42.7 (33.1;52.8)		
100-<1000 p/µL (n=106)	2.39 (1.74;3.26)			2.55 (1.82;3.55)			76.4 (67.0;83.9)		
>=1000 p/µL (n=80)	2.69 (1.94;3.70)			2.94 (2.08;4.12)			61.3 (49.7;71.7)		
**HIV**		0.38	0.47		0.21	0.35		0.77	0.80
Negative (n=220)	2.33 (2.13;2.53)			2.30 (2.11;2.51)			61.8 (49.1;73.0)		
Positive (n=69)	2.51 (1.93;3.25)			2.57 (1.98;3.32)			59.7 (52.9;66.2)		
**Gravidity**		0.36	0.64		0.043	0.44		0.11	0.79
Primi (n=117)	2.47 (2.20;2.77)			2.59 (2.31;2.89)			65.8 (56.4;74.2)		
Multi (n=172)	2.30 (1.76;3.00)			2.22 (1.70;2.88)			56.4 (48.6;63.9)		
**Season**		0.98	0.83		0.57	0.93		0.71	0.45
Dry (n=182)	2.37 (2.15;2.60)			2.41 (2.19;2.64)			59.3 (51.8;66.65)		
Rainy (n=107)	2.37 (1.84;3.04)			2.30 (1.79;2.94)			61.7 (51.7;70.8)		
**Children 2-10 years**									
**All** (n=93)	2.70 (2.38;3.05)			2.86 (2.53;3.22)			68.8 (58.8;77.3)		
**Year**		0.066	0.46		0.012	0.057		0.92	0.45
2015-2016 (n=60)	2.98 (2.57;3.44)			3.23 (2.80;3.71)			70.0 (56.6;80.8)		
2017 (n=4)	2.50 (1.06;5.16)			2.50 (1.07;5.13)			75.0 (21.9;98.7)		
2018-2019 (n=29)	2.14 (1.37;3.27)			2.14 (1,38;3.24)			65.5 (45.7;81.4)		
Change per year	-0.11 (-0.21;-0.015)	0.023	0.38	-0.16 (-0.26;-0.066)	**0.00085**	0.027	NA	NA	0.27
**Area**		0.21	0.48		0.092	0.18		0.65	0.15
Magude (n=47)	2.64 (2.20;3.13)			2.91 (2.45;3.43)			68.1 (52.7;80.5)		
Manhiça (n=42)	2.88 (1.87;4.39)			2.95 (1.95;4.43)			73.8 (57.7;85.6)		
I. Josina (n=4)	1.50 (0.49;3.70)			1.25 (0.37;3.23)			25.0 (1.32;78.1)		
**Parasite density**		0.21	0.19		**0.0011**	**0.00076**		0.57	0.68
<100 p/µL (n=33)	2.36 (1.88;2.93)			2.27 (1.80;2.83)			66.7 (48.1;81.4)		
100-<1000 p/µL (n=28)	2.86 (1.66;4.84)			3.00 (1.74;5.10)			78.6 (58.5;91.0)		
>=1000 p/µL (n=20)	3.15 (1.79;5.43)			4.10 (2.37;6.98)			75.0 (50.6;90.4)		

Naive offset multiplicity of infection (MOI) is defined as the second highest number of distinct alleles observed within one sample. MOI is estimated using MOIRE (R package). Means and 95% confidence intervals (CI) obtained from 0-truncated Poisson regression, with p-values computed with likelihood ratio tests. A sample was considered polyclonal if MOI>1. 95% CI obtained with Z test of proportions, p-values with chi square goodness-of-fit test, and adjusted p-values with likelihood ratio test in multivariate logistic regression. p = p-value for univariate analysis, p* = p-value for multivariate analysis adjusting for parasitemia (categorical), time (continuous), area, and an interaction between time and area, NA = not applicable.

Supplementary Table 2. Effect of gravidity on intra-host genetic diversity in first antenatal care users by area

	n		Log odds eMOI		% polyclonal			Log odds 1-Fws	
	primi	multi	**Multi**	**p**	**Primi**	**Multi**	**p**	**Multi**	**p**
**Magude**	54	66	-0·23 (-0·47;0·12)	0·18	55·6 (42·4;68·0)	51·5 (39·7;63·2)	0·72	-0·11 (-0·60;0·97)	0·77
**Manhiça**	28	36	-0·27 (-0·60;0·31)	0·30	53·6 (35·8;70·5)	41·7 (27·1;57·8)	0·45	-0·34 (-0·80;1·10)	0·48
**Ilha**	35	70	0·11 (-0·32;0·89)	0·68	51·4 (35·6;67·0)	51·4 (40·0;62·8)	1	0·26 (-0·51;2·50)	0·64

Within-area estimates of the effect of multigravidity on intra-host diversity from zero-truncated Poisson and logistic multivariate regressions, respectively, adjusting for parasitemia (<100, 100-<1000, >=1000). eMOI = effective multiplicity of infection, estimated with *MOIRE* (R package).[3] Means and 95% confidence intervals (CI) obtained from 0-truncated Poisson regression, with p-values computed with likelihood ratio tests (two-sided). Polyclonal infections are defined as having an eMOI>1.1. 95% CI obtained with Z test of proportions, p-values with chi square goodness-of-fit test, and adjusted p-values with likelihood ratio test in multivariate logistic regression (two-sided). $1-F_{ws}$ = within-host heterozygosity relative to population expected heterozygosity at a given allele. Means and 95% CI are obtained from logistic regression, with p-values from likelihood ratio tests (two-sided). N=120 in Magude, n=64 in Manhiça, n=105 in Ilha Josina.

Supplementary Table 3. Temporal trends in intra-host genetic diversity first antenatal care users in each area

	Change in eMOI per year (%)		Change in OR polyclonal per year		Change in 1-Fws per year (%)	
	(95% CI)	p	(95% CI)	p	(95% CI)	p
**Magude**	-0·50 (-0·78;-0·25)	**0·0002**	-0·46 (-0·98;0·03)	0·071	-0·58 (-1·16;-0·05)	0·037
**Manhiça**	0·17 (-0·18;0·52)	0·33	0·06 (-0·58;0·70)	0·86	0·14 (-0·58;0·86)	0·69
**Ilha Josina**	0·06 (-0·27;0·36)	0·72	0·63 (0·07;1·24)	0·033	0·22 (-0·39;0·79)	0·46

Within-area estimates of annual change in intra-host diversity from zero-truncated Poisson and logistic multivariate regressions, respectively, adjusting for parasitemia (<100, 100-<1000, >=1000). p for temporal trend in multivariate models with a continuous time variable (two-sided). eMOI = effective multiplicity of infection, estimated with *MOIRE* (R package).[3] Polyclonal infections are defined as having an eMOI>1.1. F_{ws} = within-host heterozygosity relative to population expected heterozygosity at a given allele. N=120 in Magude, n=64 in Manhiça, n=105 in Ilha Josina. Multiple testing weas corrected for using the Benjamin-Hochberg procedure.

Supplementary Table 4. Differences in temporal trends in Magude genetic intra-host diversity between first antenatal care users and children

	n	Change in eMOI per year (95% CI)	p	p*	Change in OR for polyclonality per year (95% CI)	p	p*	Change in 1-Fws per year (95% CI)	p	p*
**Children**	47	-0·37 (-0·58;-0·18)	**0·0003**	0·20	-0·001 (-0·43;0·42)	1.00	0·15	-0·20 (-0·65;0·22)	0·36	0·53
**ANC**	120	-0·51 (-0·78;-0·25)	**0·0002**		-0·46 (-0·97;0·04)	0·062		-0·59 (-1·17;-0·05)	0·037	

Estimates of annual change in intra-host diversity among children aged 2-10 years and pregnant women attending their first antenatal care (ANC) visit, adjusting for parasitemia (<100, 100-<1000, >=1000). p-value for temporal trend (two-sided). *p-value for difference between populations (two-sided). eMOI = effective multiplicity of infection, estimated with *MOIRE* (R package).[3] Polyclonal infections are defined as having an eMOI>1.1. F_{ws} = within-host heterozygosity relative to population expected heterozygosity at a given allele. Multiple testing was corrected for using the Benjamin-Hochberg procedure.

Supplementary table 5. Correlation between trends in genetic intra-host diversity and *P. falciparum* parasite rates in first antenatal care users by area

Area	Genetic metric	PCC	p
**Magude**	**eMOI**	0.90	0.015
	**Polyclonal**	0.68	0.21
	**1-Fws**	0.79	0.062
**Manhiça**	**eMOI**	-0.53	0.28
	**Polyclonal**	-0.37	0.47
	**1-Fws**	-0.51	0.30
**Ilha Josina**	**eMOI**	-0.086	0.87
	**Polyclonal**	-0.59	0.22
	**1-Fws**	-0.33	0.53

Correlation between metrics of genetic intra-host diversity and *P. falciparum* parasite rates by qPCR in 6-month time bins analyzed with Pearson correlation test (two-sided). PCC = Pearson correlation coefficient. eMOI = effective multiplicity of infection. Polyclonality = eMOI >1.1. 1-Fws = within-host heterozygosity relative to population expected heterozygosity at a given allele. N=120 in Magude, n=64 in Manhiça, n=105 in Ilha Josina. Multiple testing was corrected for using the Benjamin-Hochberg procedure.

Supplementary table 6. Correlation between trends in genetic intra-host diversity and *P. falciparum* parasite rates in children in Magude

	PCC	p
**eMOI**	0.86	0.064
**Polyclonal**	-0.31	0.62
**1-Fws**	0.35	0.57

Correlation between metrics of genetic intra-host diversity and *P. falciparum* parasite rates by qPCR per year analyzed with Pearson correlation test (two-sided). PCC = Pearson correlation coefficient. eMOI = effective multiplicity of infection. Polyclonality = eMOI >1.1. 1-Fws = within-host heterozygosity relative to population expected heterozygosity at a given allele. N=47. Multiple testing was corrected for using the Benjamin-Hochberg procedure.

Supplementary Table 7. Expected heterozygosity in first antenatal care users by area and years

	n	Overall mean H_E (95% CI)	p	n	2017 mean H_E (95% CI)	n	2018-2019 mean H_E (95% CI)	p
**All**	289	0·57 (0·54;0·60)		123	0·56 (0·53;0·59)	123	0·56 (0·52;0·60)	0·96
**Magude**	64	0·53 (0·50;0·56)	**0·0012**	58	0·54 (0·51;0·57)	58	0·53 (0·49;0·57)	0·15
**Manhiça**	64	0·54 (0·50;0·58)		31	0·50 (0·47;0·53)	31	0·52 (0·48;0·56)	**0·0022**
**Ilha**	64	0·55 (0·51;0·59)		28	0·50 (0·47;0·53)	28	0·51 (0·47;0·56)	0·22

Expected heterozygosity (H_E), i.e., the probability that two randomly selected parasites carry distinct alleles at each diversity locus (n=165), was estimated with MOIRE. H_E was compared between populations with Linear Mixed Models (R package *nlme*) fitting locus as a random effect. P-value from F-test. A simple random subsampling of the larger populations was done to match the smaller population in size. Multiple testing was corrected for using the Benjamin-Hochberg procedure.

Supplementary Table 8. Expected heterozygosity in first antenatal care users and children

	n	Overall mean H_E (95% CI)	p
**ANC**	33	0·50 (0·49;0·52)	0·95
**Community**	33	0·50 (0·47;0·53)	

Expected heterozygosity (H_E), i.e., the probability that two randomly selected parasites carry distinct alleles at each diversity locus (n=165), was estimated with MOIRE. H_E was compared between populations with Linear Mixed Models (R package *nlme*) fitting locus as a random effect. P-value from F-test. A simple random subsampling of the ANC populations was done to match the children population in size, area, and year.

Supplementary Table 9. Pairwise relatedness (identity by descent) within and between areas for first antenatal care users

	Magude (n pairs)	p	Manhiça (n pairs)	p	Ilha Josina (n pairs)	p
**Magude**	0·031 (14,280)	**0·0020**	0·025 (7,680)	0·72	0·025 (12,600)	0·73
**Manhiça**	0·025 (7,680)	0·72	0·024 (4,032)	0·69	0·024 (6,700)	0·96
**Ilha**	0·025 (12,600)	0·73	0·024 (6,700)	0·96	0·025 (10,930)	0·71

Identity by descent (IBD), i.e., pairwise relatedness between infections, was estimated with *Dcifer* (R package).[4] All infections, including unphased polyclonal ones, are included. To test for differences in mean IBD between and within areas, we performed permutation of area labels (10,000 permutations) and compared mean IBD with permutation distributions. P-values are from two-sided permutation tests. Overall mean relatedness between ANC infection pairs was 0.026 (96% CI: 0.022;0.033). Multiple testing was corrected for using the Benjamin-Hochberg procedure.

Supplementary Table 10. Pairwise relatedness (identity by descent) between infections in pregnant women attending antenatal care and children overlapping in time

	**ANC** (n=39)	**p**	**Community** (n=31)	**p**
**ANC** (n=39)	0·018	0·30	0·017	0·67
**Community** (n=31)	0·017	0·67	0·017	0·54

Identity by descent (IBD), i.e., pairwise relatedness between infections, was estimated with *Dcifer* (R package).[4] All infections, including unphased polyclonal ones, are included. To test for differences in mean IBD between and within areas, we performed permutation of group labels (10,000 permutations) and compared mean IBD with permutation distributions. P-values are from permutation tests. Multiple testing was corrected for using the Benjamin-Hochberg procedure.

References

1 Pujol, A. *et al.* Detecting temporal and spatial malaria patterns from first antenatal care visits. *Nature Communications* **14**, 4004 (2023). https://doi.org:10.1038/s41467-023-39662-4

2 Galatas, B. *et al.* A multiphase program for malaria elimination in southern Mozambique (the Magude project): A before-after study. *PLOS Medicine* **17**, e1003227 (2020). https://doi.org:10.1371/journal.pmed.1003227

3 Murphy, M. & Greenhouse, B. MOIRE: A software package for the estimation of allele frequencies and effective multiplicity of infection from polyallelic data. *bioRxiv*, 2023.2010.2003.560769 (2023). https://doi.org:10.1101/2023.10.03.560769

4 Gerlovina, I., Gerlovin, B., Rodríguez-Barraquer, I. & Greenhouse, B. Dcifer: an IBD-based method to calculate genetic distance between polyclonal infections. *Genetics* **222** (2022). https://doi.org:10.1093/genetics/iyac126
