# Supplementary material for: Genomic malaria surveillance of antenatal care users detects reduced transmission following elimination interventions in Mozambique
Source: Nat Commun. 2024 Mar 16;15:2402. doi: 10.1038/s41467-024-46535-x (PMC10944499; doi:10.1038/s41467-024-46535-x)
Supplement: Supplementary file 3 — Description of Additional Supplementary Files [file 41467_2024_46535_MOESM3_ESM.pdf]

**Description of Additional Supplementary Files**

File Name: Supplementary Data 1
Description: Primers used for amplicon sequencing
