# Supplementary material for: Genomic malaria surveillance of antenatal care users detects reduced transmission following elimination interventions in Mozambique
Source: Nat Commun. 2024 Mar 16;15:2402. doi: 10.1038/s41467-024-46535-x (PMC10944499; doi:10.1038/s41467-024-46535-x)
Supplement: Supplementary file 5 — Reporting Summary [file 41467_2024_46535_MOESM5_ESM.pdf]

nature portfolio

Corresponding author(s): Alfredo Mayor

Last updated by author(s): Feb 20, 2024

Reporting Summary

Nature Portfolio wishes to improve the reproducibility of the work that we publish. This form provides structure for consistency and transparency in reporting. For further information on Nature Portfolio policies, see our Editorial Policies and the Editorial Policy Checklist.

Statistics

For all statistical analyses, confirm that the following items are present in the figure legend, table legend, main text, or Methods section.

n/a	Confirmed	
☐	☒	The exact sample size (*n*) for each experimental group/condition, given as a discrete number and unit of measurement
☐	☒	A statement on whether measurements were taken from distinct samples or whether the same sample was measured repeatedly
☐	☒	The statistical test(s) used AND whether they are one- or two-sided<br>*Only common tests should be described solely by name; describe more complex techniques in the Methods section.*
☐	☒	A description of all covariates tested
☐	☒	A description of any assumptions or corrections, such as tests of normality and adjustment for multiple comparisons
☐	☒	A full description of the statistical parameters including central tendency (e.g. means) or other basic estimates (e.g. regression coefficient) AND variation (e.g. standard deviation) or associated estimates of uncertainty (e.g. confidence intervals)
☐	☒	For null hypothesis testing, the test statistic (e.g. *F*, *t*, *r*) with confidence intervals, effect sizes, degrees of freedom and *P* value noted<br>*Give P values as exact values whenever suitable.*
☐	☒	For Bayesian analysis, information on the choice of priors and Markov chain Monte Carlo settings
☒	☐	For hierarchical and complex designs, identification of the appropriate level for tests and full reporting of outcomes
☐	☒	Estimates of effect sizes (e.g. Cohen's *d*, Pearson's *r*), indicating how they were calculated

Our web collection on statistics for biologists contains articles on many of the points above.

Software and code

Policy information about availability of computer code

Data collection	A standardised electronic questionnaire using REDCap was filled in. The application is described at https://projectredcap.org/.
Data analysis	Fastq files were processed with a Nextflow-based pipeline available at https://github.com/EPPIcenter/mad4hatter, version 0.1.5. R version 4.3.0 was used to analyze the resulting tables of alleles and drug resistance marker. Genetic diversity was estimated using the R package MOIRE version 3.0.0, available at https://github.com/EPPIcenter/moire and in a zip-file enclosed to editors. MOIRE is in the process of peer-review, with the preprint available at https://www.biorxiv.org/content/10.1101/2023.10.03.560769v2.full. Expected heterozygosity was compared between groups using linear mixed models with the R package nlme version 3.1, described in https://cran.r-project.org/web/packages/nlme/nlme.pdf. Genetic relatedness was estimated using the R package Dcifer version 1.2.0, available at https://github.com/EPPIcenter/dcifer.

For manuscripts utilizing custom algorithms or software that are central to the research but not yet described in published literature, software must be made available to editors and reviewers. We strongly encourage code deposition in a community repository (e.g. GitHub). See the Nature Portfolio guidelines for submitting code & software for further information.

Data

Policy information about availability of data

All manuscripts must include a data availability statement. This statement should provide the following information, where applicable:

- Accession codes, unique identifiers, or web links for publicly available datasets
- A description of any restrictions on data availability
- For clinical datasets or third party data, please ensure that the statement adheres to our policy

Sequencing data and some epidemiological meta data is available at NCBI Sequence Read Archive (SRA) under accession code PRJNA1040019 [https://www.ncbi.nlm.nih.gov/bioproject/PRJNA1040019]. Sequencing data was aligned to selected regions of the Pf3D7 genome [https://github.com/EPPIcenter/mad4hatter/blob/main/resources/v4/ALL_refseq.fa.] The raw epidemiological data includes personal data and is protected by data privacy laws. This data can be made available for research purposes from the corresponding author, AM, upon reasonable request by email. Requests for data will be reviewed in a three-month timeframe by Manhiça Health Research Center to verify that data sharing is not subject to any intellectual property or confidentiality obligations. If data can be shared, it will be released via a Data Transfer Agreement.

Research involving human participants, their data, or biological material

Policy information about studies with human participants or human data. See also policy information about sex, gender (identity/presentation), and sexual orientation and race, ethnicity and racism.

Reporting on sex and gender	Study participants recruited from antenatal care clinics were all pregnant and thus biological women by definition. Study participants recruited in household surveys were selected to be representative of the general population. Self-reported gender was evenly represented among the children aged 2-10 years old in the survey, with 50.3% girls and 49.2% boys (unavailable information for remaining 0.5%). We did not stratify the analysis by sex/gender, as it was not relevant for the pregnant women, while for the children, the sample size did not permit this, nor did we expect it to affect our results.
Reporting on race, ethnicity, or other socially relevant groupings	No socially constructed or socially relevant groupings were used in the analysis.
Population characteristics	Population characteristics are described in Table 1. Briefly, pregnant women recruited at antenatal care had a mean age of 21 years (95% confidence interval 18-27), a geometric mean parasite density of 86.6 parasites/microliter, most of them were multigravid (235/378), HIV-negative (291/378), and sampled during dry season (233/378). Children recruited in cross-sectional household surveys had a mean age of 4 years (95% confidence interval 3.0-6.3), a geometric mean parasite density of 41.1 parasites/microliter, and were all sampled during dry season.<br>More detailed characteristics of the pregnant women attending antenatal care can be found in https://doi.org/10.1186/s12916-022-02597-6.
Recruitment	Pregnant women were approached and invited to participate in the study at their first routine ANC visits. Inclusion criteria was residing in the study area and being willing to participate. Eligible women were read an informed consent statement by trained maternity nurses or midwives, and their consent was subsuquently obtained. Study participants were asked to donate a fingerprick blood drop onto filter papers and a brief form was filled out, including information on visit date, age, gravidity, gestational age based on the fundal height measurement, area of residence, and recent movements. HIV status was recorded from the maternal health card. In case of an unavailable record, an HIV serological rapid test was done according to standard procedures for voluntary counseling and testing.<br>For the cross-sectional surveys, a random age-stratified sample of the population of Manhiça (180,000 individuals) and Magude (60,000 individuals) districts, with oversampling of children under 15 years old, was selected from the Centro de Investigação em Saúde de Manhiça (CISM) Demographic Health Surveillance System (DHSS) census. A standardised electronic questionnaire using REDCap was completed for each participant with basic socio-demographic, clinical and vector control information. From all consenting participants finger-prick blood samples were collected for malaria diagnostics by microscopy, rapid diagnostic test (RDT) and qPCR. The surveys were conducted around May each year.<br>The study population may be affected by self-selection bias if decision to participate is associated was associated with risk factors for malaria. We would expect participating individuals to generally be more compliant with health care providers, and might therefore have a higher usage of preventive measures such as bed nets, and thus a decreased risk of malaria exposure. However, participation rate was high (85%), and self-selection bias is therefore expected to be minimal. Selection bias might also arise from the decision to attend ANC. In general, women not attending ANC tend to be older, live in rural settings, and have lower socio-economic status than ANC attending women, which are all risk factors for malaria. Thus, ANC-attending women would be expected to have lower malaria risk. However, this selection bias is likely to be low in sub-Saharan Africa due to high ANC attendance, and in any case the goal of the study is to assess the potential of malaria surveillance using data from antenatal care visits, so the bias would be expected to be present in an ANC-based surveillance system.
Ethics oversight	Study protocols were approved by Manhiça Health Research Centre's and Hospital Clínic of Barcelona's ethics committees, and the Mozambican Ministry of Health National Bioethics Committee.

Note that full information on the approval of the study protocol must also be provided in the manuscript.

Field-specific reporting

Life sciences study design

All studies must disclose on these points even when the disclosure is negative.

Sample size	Pregnant women at first antenatal care visit:<br>The sample size of pregnant women recruited at antenatal care was calculated to be able to detect malaria prevalence of 10% in Manhiça, 45% in Ilha Josina, and <10% in Magude with 1-2% accuray, taking local attendance rates into account. It was assumed that 10% of women would decline or not be eligible to enroll. In total, the aim was to enroll 1800, 270 and 900 pregnant women in Manhiça, Ilha Josina, and Magude, respectively, per year. For the qPCR detection of Pf in pregnant women, all samples collected from Ilha Josina were analyzed (n=250 per year), while a random selection of approximately 2700 samples per year in both Magude and Manhiça was used to allow for the estimation of the 95% confidence interval of annual P. falciparum positivity rates between 20% and 5% in each of the 3 sites, with a margin of error lower than or equal to the expected positivity rate. For amplicon sequencing, a total of 378 samples from pregnant women were available for sequencing.<br><br>Children from cross-sectional surveys:<br>For Manhiça district, the sample size was estimated to detect a prevalence of malaria of 50% (conservative estimate) with a precision of ±10% approximately and a 95% confidence interval per age group. For Magude district, sample sizes were calculated based on the two sampling scenarios: a) Simple random sampling: a sample of 384 participants per age group (980 in total) were needed to detect a malaria prevalence of 50%, with a precision of ±5% and a 95% level of confidence. b) Multi-stage cluster random sampling: due to the logistical and resource constrains that would involve sampling a group large enough to detect the most conservative prevalence with maximum precision using multi-stage cluster random sampling, in this case, the sample size calculations were set to detect a prevalence of 20%, with a precision of ±10% and a 95% level of confidence, assuming a design effect of 2, a 10% non-response rate, and an average administrative post size of 50 people. For amplicon sequencing, a total of 180 samples from children aged 2-10 years old were available for sequencing.
Data exclusions	Data exclusion criteria was pre-established before the study. Individuals were excluded if they did not consent to participate, and if they did not live within the study area, moved during the study period, or if information about HIV-status was not available. Data from antenatal care visits other than the first were excluded due to potential seleciton of parasite genotypes due to intermittent preventive treatment administered.<br>All Pf-positive available sample were sequencing. Samples with low read counts were re-sequenced at higher proportions, and if still not succesful, were excluded from the analysis. Exclusion of reads were done with cutadapt and DADA2 (see methods section) based on quality and length. Subsequently, alleles with fewer reads than the maximum observed reads in any locus for negative controls (14 reads) were removed, along with alleles with <1% within-sample frequency. Samples with a coverage of <50 diversity loci with a read depth of 100 were filtered out. Finally, diversity loci with <100 samples covering them with a read depth of 100 were also removed.
Replication	DNA quantification was repeated for a subset of samples (~10%) in another lab to confirm that results were generally replicable. Furthermore, a subset of samples (~5%) were re-sequenced and data analysis was repeated, confirming the replicability of findings on multiplicity of infection.
Randomization	Sample collection was carried out separately at antenatal care clinics and during household surveys, so this process could not be randomized. For sequencing, dried blood spots were organized based on parasite density (because the library preparation protocol depended on this), and were randomized with regards to other characteristics including population group, area of residence etc.
Blinding	Investigators were not blinded during data collection, because the site and procedure differed between population groups (pregnant women recruited at antenatal care visits and children recruited for household surveys). Investigators were blinded to characteristics other than parasite densities during lab procedures, including population group, area of residence etc.

Reporting for specific materials, systems and methods

We require information from authors about some types of materials, experimental systems and methods used in many studies. Here, indicate whether each material, system or method listed is relevant to your study. If you are not sure if a list item applies to your research, read the appropriate section before selecting a response.

Materials & experimental systems

n/a	Involved in the study
☒	☐ Antibodies
☒	☐ Eukaryotic cell lines
☒	☐ Palaeontology and archaeology
☒	☐ Animals and other organisms
☒	☐ Clinical data
☒	☐ Dual use research of concern
☒	☐ Plants

Methods

n/a	Involved in the study
☒	☐ ChIP-seq
☒	☐ Flow cytometry
☒	☐ MRI-based neuroimaging

Plants

Seed stocks

Report on the source of all seed stocks or other plant material used. If applicable, state the seed stock centre and catalogue number. If plant specimens were collected from the field, describe the collection location, date and sampling procedures.

Novel plant genotypes

Describe the methods by which all novel plant genotypes were produced. This includes those generated by transgenic approaches, gene editing, chemical/radiation-based mutagenesis and hybridization. For transgenic lines, describe the transformation method, the number of independent lines analyzed and the generation upon which experiments were performed. For gene-edited lines, describe the editor used, the endogenous sequence targeted for editing, the targeting guide RNA sequence (if applicable) and how the editor was applied.

Authentication

Describe any authentication procedures for each seed stock used or novel genotype generated. Describe any experiments used to assess the effect of a mutation and, where applicable, how potential secondary effects (e.g. second site T-DNA insertions, mosiacism, off-target gene editing) were examined.
